# Determinants of Open Defecation in the Wa Municipality of Ghana: Empirical Findings Highlighting Sociocultural and Economic Dynamics among Households

**DOI:** 10.1155/2019/3075840

**Published:** 2019-02-03

**Authors:** Issaka Kanton Osumanu, Enoch Akwasi Kosoe, Frank Ategeeng

**Affiliations:** Department of Environment and Resource Studies, University for Development Studies, Tamale, Ghana

## Abstract

This study examined sociocultural and economic factors determining open defecation in the Wa Municipality, Ghana. The study employed a mixed method approach involving questionnaire administration to 367 households systematically selected from 21 communities, observation, and eight key informant interviews. The mixed logit model was used to determine the factors that significantly influence open defecation. The findings revealed that 49.8% of households had no form of toilet facility at home and were either using communal/public toilets or practicing open defecation. Several sociocultural and economic reasons account for this. But for these households, having a toilet facility at home does not seem to be a priority. Six factors (education, household size, occupation, income, traditional norms, and beliefs and ownership of a toilet facility) were positively significant in determining open defecation. Fundamental to many of the significant factors is households' capacity to finance construction of home toilets. In addition to finding new and innovative approaches to public education, the principle of credit financing, that incorporates community-led initiatives, may be considered in assisting households to construct home toilets.

## 1. Introduction

Open defecation continues to be a critical health challenge globally, affecting almost 1 billion people worldwide and contributing significantly to an estimated 842,000 people who die yearly from sanitation-related diseases [1]. Open defecation is a major environmental health problem facing many countries in sub-Saharan Africa. Although open defecation rates have been reducing gradually since 2000, the Millennium Development Goals (MDGs) era ended without all countries in sub-Saharan Africa achieving target 7.C., which included reducing by half the proportion of the population without sustainable access to basic sanitation by 2015 [2]. Some estimates indicate that, at the current rate, this can only be achieved by 2026 [3]. All sustainable development goal (SDG) regions saw a drop in the number of people practicing open defecation, except for sub-Saharan Africa, where high population growth led to an increase in open defecation from 204 million to 220 million, and in Oceania, where the practice increased from 1 million to 1.3 million [4]. This information is supported by studies such as Osumanu and Kosoe [5], which shows that open defecation in Ghana has increased over the years resulting in several environmentally endemic health problems.

The existence of open defecation is associated with diseases, under nutrition and poverty, and is usually considered as an affront to personal dignity. Those countries where open defecation is most widely practiced have the highest numbers of deaths of children under the age of five, as well as high levels of under nutrition, high levels of poverty, and large disparities between the rich and poor [3]. In sub-Sahara Africa, 200,000 children under the age of five die from diarrhoea annually, while the numbers dying from cholera within the region are similarly high because of poor sanitation, hygiene practices, and unsafe water supplies [1].

Ghana has been ranked second after Sudan in Africa for open defecation, with almost 5 million Ghanaians not having access to any toilet facility [3]. The number of people practicing open defecation in Ghana was reported at 18.75% in 2015 [4]. This refers to the percentage of the population defecating in the open, such as in fields, forests, bushes, open bodies of water, on beaches, and in other open spaces or those who dispose human excreta with solid waste. The practice is most prevalent in the Upper East Region with about 89% of the people without any form of latrine, followed by the Northern Region with about 72% and the Upper West Region with about 71% [3]. Households which have no toilet facilities of any kind available for use at home in the Upper West Region mostly resort to use of the bush or the field or small receptacles that are available for defecation [6]. The seriousness of the situation is that children are the leading culprits, particularly because toilet facilities are usually not designed to meet their needs [7].

Though open defecation is predominantly a rural phenomenon, it is estimated that 8.22% of the urban population in sub-Saharan Africa practice open defecation [4]. The practice of open defecation in urban areas needs attention because research has shown that detrimental health impacts (particularly for early life health) are more significant from open defecation when the population density is high. For instance, the same amount of open defecation is twice as bad in a place with a high population density average vis-à-vis a low population density average [8].

Open defecation is increasingly becoming alarming in the Wa Municipality, putting residents at the risk of sanitation related diseases such as cholera, diarrhoea, and typhoid. A recent study by Kosoe and Osumanu [7] has shown that 52% of households in the Wa Municipality do not have any toilet facility at home and resort to the use of available bushes, uncompleted buildings, and open spaces within their neighbourhoods. Human faeces are found in open spaces and in-between houses, some rapped in black polythene bags, with the resultant stench and flies nuisance. The sight and smell of faeces within residential neighbourhoods reduce the aesthetic quality of the environment and causes embarrassment to residents and visitors to the municipality.

Open defecation practices in every society are surrounded by sociocultural and economic factors, which must be well understood before any sanitation programme can hope to be effective [6]. The major objective of this study is to examine the socioeconomic and cultural factors determining open defecation in the Wa Municipality. The examination is done within the context of open defecation practices across the entire municipality (both rural and urban). Unlike earlier studies [5, 6], this study provides a comprehensive quantitative examination of the determinants of open defecation based on identified sociocultural and economic factors. The main contribution of this paper is analysis of significant determinants of open defecation in the Wa Municipality. This will assist in targeting appropriate specific open defecation elimination strategies to ameliorate its health implications in the Municipality and Ghana at large. The paper is categorized into six subsections consisting of introduction, conceptual framework, description of the study area and methodology, results, discussion, and conclusion and policy implications.

## 2. Determinants of Open Defecation: Towards a Conceptual Framework

**Figure 1:**
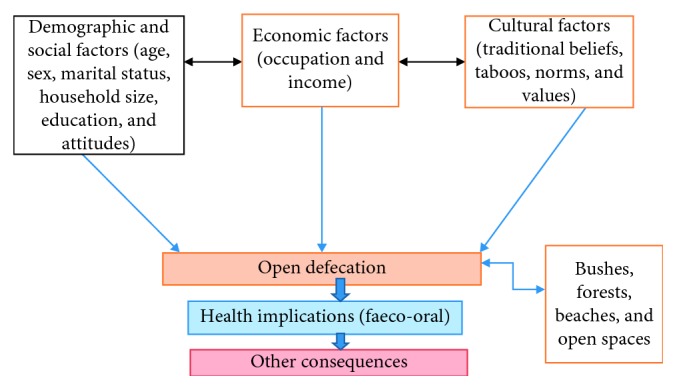
A conceptual framework.

Open defecation is an old sanitation issue globally, and in developing countries in particular, which persist till date despite its damning effects. Why the practice continues to persist is a question that remains largely unanswered. The conceptual framework represented in Figure 1 is a synthesis of literature and attempts to provide an understanding of how the practice of open defecation is connected with identified sociocultural and economic factors.

The technical feasibility and acceptability of a particular sanitation system depends on several factors including cost and affordability as well as communal or household characteristics. There is a relationship between household wealth/social status and latrine ownership/open defecation. Improved latrine owners are wealthier than unimproved latrine owners or open defecators; are more educated; and have higher literacy rates [3, 9]. In general, low-income groups do not spend more than 2–5% of their income on excreta disposal [10]. Osumanu and Kosoe [5] contend that financial constraints present two challenges. First, it inhibits house owners from the provision of household toilets, and secondly, it causes people's inability to afford fees charged by public toilet operators. This implies that if a household cannot afford the fees for the use of a public toilet and cannot also afford to construct a toilet facility, they will practice open defecation.

Water Aid [6] identified negative attitudes involving carelessness, disrespect for traditional authority, and community norms as determinants of open defecation, which suggest ineffectiveness of the law and order. Also, according to Jenkins and Scott [11], the adoption of latrines in poor communities follows three behavioural patterns: preference, intention, and choice. The third pattern, choice, is however based on the financial standing of the individual. Social norms, the rules that govern how individuals in a group or society behave including behavioural standards that exist in the community for an individual to follow and the presence or absence of traditions and cultures that govern behaviour [9, 12], are also contributory factors to open defecation. Family members, peers, and others in the community defecate in the open, making this a common behaviour that is rooted in culture and tradition and learned since childhood. Connell [9] observed that in Peru, open defecation is described as “*the most natural thing*,” and he described the practice as traditional, habitual, and part of one's daily routine and that these social norms are also held more strongly by open defecators.

Belcher [13] reported that in Uganda, in the late 1940s, people were afraid to use latrines because their fixed location would provide sorcerers with easy access to their excreta for devilish purposes; and faeces of one's own in contact with another could bring about “*spiritual contamination*,” hence defecating at random in the bush and surroundings was considered a safer alternative. The importance of traditional beliefs and perceptions in latrine use and open defecation was also amply demonstrated in Kumasi, Ghana, when Cotton et al. [14] reported that a householder refused to use a latrine because he was a Muslim and the latrine faced the direction of Mecca. Similar superstitious traditional beliefs have been reported by Water Aid [6] in some communities in Burkina Faso and Mali as well as Tamale (Ghana), and for the Idoma people in Nigeria, and by Osumanu and Kosoe [5] in Wa (Ghana).

## 3. Study Context and Methodology

**Figure 2:**
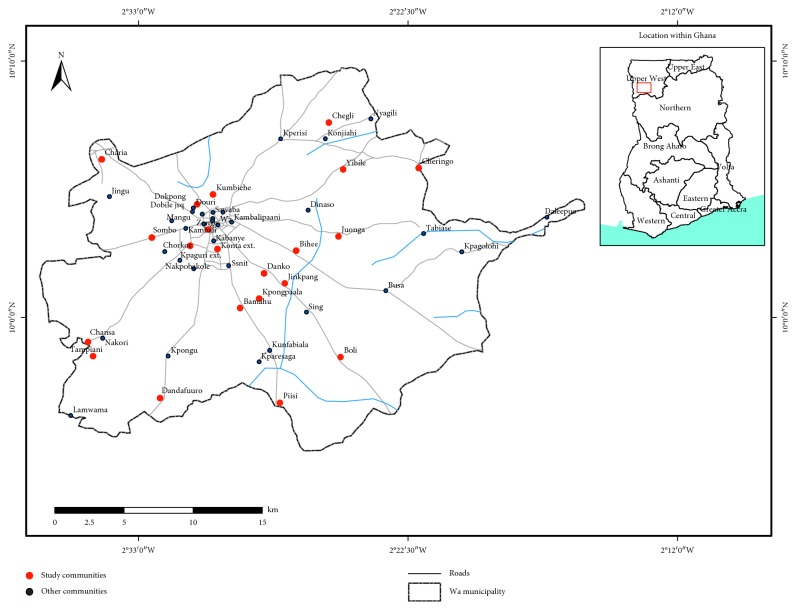
Map of Wa Municipality showing the study communities. Source: adapted from Wa Municipal Assembly (2013).

The locational context of this study is Wa Municipality. Wa Municipality lies between latitude 10°40′N and 20°45′N and on longitude 90°32′W (Figure 2), thus covering an area of approximately 1,180 square kilometres which is about 32% and 2.56% of the Upper West Region and Ghana, respectively. According to Ghana Statistical Service [15], the total population of the Wa Municipality is 107,214, which constitutes 15.3% of the population of Upper West Region.

This study employed a descriptive and interpretive case study design [16], which permits an in-depth investigation of individuals, groups, or events which may be descriptive or explanatory. The mixed method approach was used to collect data for the study. The mixed method helps in inferring both qualitative and quantitative data in a single study or in sequential studies based on priority and sequence of information [17]. Questionnaires were used to collect data for the quantitative study. The qualitative methods that were used include observations and interviews. In-depth interviews were carried out with specific participants from the Wa Municipal Assembly, the Municipal Directorate of the Ghana Health Service, and the Community Water and Sanitation Agency, as well as religious and traditional bodies.

Both probability and nonprobability sampling techniques were employed in the selection of respondents. Simple random sampling technique was used in selecting 21 out of the 84 communities within the five Administrative Zonal Councils in the Wa Municipality, and systematic random sampling used to select housing units for questionnaire administration to the various households. Blocks were created in the selected communities based on the number of houses in the community, and interviewers selected households to interview by systematically walking through the blocks and interviewing household heads of selected housing units or their representatives. In a house where there were multiple households, only one household head was interviewed. Again in each selected house where the household interviewed was not the owner of the house, an attempt was made to interview the owner of the house (the landlord).

The number of households in all the selected communities (*N* = 4,475) was obtained from Ghana Statistical Service [15] and used for the study. Yamane's [18] formula for determining sample size, that is *n* = *N*/(1 + *N* (*α*)^2^), where *n* = sample size, *N* = sample frame, and *α* is the margin of error estimated at 0.05, was used to determine the sample size. This yielded a total sample of 367 households. The allocation of sample sizes to each community was influenced by the number of households in each community for purposes of achieving representation. Simple proportions were used to allocate the total sample to the 21 selected communities as shown in Table 1.

Purposive sampling was used to select those sample units that are directly in authority in managing sanitation in the Wa Municipality and leaders of recognised religious and traditional bodies. These categories of respondents formed the key informants for the study. In all, eight key informants, comprising the Wa Municipal Environmental Health and Sanitation Officer, the Municipal Disease Control Officer of the Ghana Health Service, the Regional Director of the Community Water and Sanitation Agency, two public toilet attendants, one Christian leader, one Muslim leader, and one Traditional leader.

The information obtained in quantitative form was transformed into descriptive statistics involving frequency counts, means, and percentages for purposes of analyses and interpretation. Qualitative information was analysed manually using content analysis. Content analysis refers to “a variety of techniques for making inferences by objectively and systematically identifying specified characteristics of messages” [13, 19]. Topic coding was used to group the texts into various categories in accordance with the subthemes of this study. The categories identified pertained to the sociocultural and economic factors which influence open defecation. The mixed logit model [20] was used for analysis of determinants of open defecation. This model is considered as the most promising state-of-the-art discrete choice model currently in use [21]. Theoretically, the logit model is specified as follows:

(1) $$\begin{array}{c} Y_{i}=X_{i}+{µ}_{i}, \end{array}$$

where *Y* = *a* dummy variable such that *Y* = 1 if the factor is significant in influencing open defecation and *Y* = 0 if otherwise. *X*_*i*_ is a vector of sociocultural/economic variables.

Also,

(2) $$\begin{array}{c} \text{Prob}\left(Y=1\;|\;x\right)=f\left(x,\beta \right), \end{array}$$

(3) $$\begin{array}{c} \text{Prob}\left(Y=0\;|\;x\right)=1-f\left(x,\beta \right), \end{array}$$

where *x* is a vector of variables influencing open defecation.

According to Hensher and Greene [20], a suitable function can be adopted for the right-hand side of equations (2) and (3), such that *f*(*x*, *β*) = *x*^1^*β*. Since *E*(*Y* ∣ *x*) = *f*(*x*, *β*), the regression model can be constructed as follows:

(4) $$\begin{array}{c} Y=E\left(Y\;|\;x\right)+\left[Y-E\left(Y\;|\;x\right)\right]=x^{1}\beta +\varepsilon . \end{array}$$

The estimation of such a linear model cannot be assured that the predictions from this model will truly look like probabilities. Thus *x*^1^*β* can be constructed as the (0-1) interval. The requirement then is a model that will produce predictions consistent with (3) and (4). For a given regression vector, it could be expected that

(5) $$\begin{array}{c} \lim\text{Prob}\left(Y=1\;|\;x\right)=1,\\ {\text{x}}^{1}\beta ⟶+\infty , \end{array}$$

(6) $$\begin{array}{c} \lim\text{Prob}\left(Y=1\;|\;x\right)=0,\\ x^{1}\beta ⟶-\infty . \end{array}$$

From the above, the logistic regression model is given as

(7) $$\begin{array}{c} \text{Prob}\left(Y=1\;|\;x\right)=e^{x^{1}\beta }=\Phi \left(x^{1}\beta \right),\\ 1+e^{x^{1}\beta }, \end{array}$$

where Ф is the logistic cumulative distribution.

(8) $$\begin{array}{c} \text{Participation}=\beta_{0}+\beta_{1}\text{Age}+\beta_{2}\text{Sex}+\beta_{3}\text{Household size}+\beta_{4}\text{household position}+\beta_{5}\text{Education}+\beta_{6}\text{Occupation}+\beta_{7}\text{Group}+\beta_{8}\text{Residential status}+\beta_{9}\text{Community role}+\beta_{10}\text{Local taboo}+\beta_{11}\text{Ownership of reserve}+\beta_{12}\text{Economic}\;\;+\beta_{13}\text{Environmental}+\beta_{14}\text{Managerial benefit}+e. \end{array}$$

The outcome variable for the study was influencing open defecation. This was entered as a dummy variable indicating “Yes” or “No” and coded 1 and 0, respectively. The predictor variables are shown in Table 2.

## 4. Results

### 4.1. Background Characteristic of Respondents

Respondents' sociodemographic and economic characteristics covered by the study are sex, age, marital status, household size, level of education, occupation, and income (as shown in Table 3). From the data, the majority (61.9%) of the respondents were males. Though the respondents were systematically selected, this result reflects the dominance of males as household heads in the Wa Municipality. Similarly, and like it is in northern Ghana, males are traditionally responsible for household and community decisions including the provision of sanitation facilities. The age of respondents ranged from 19 to 70 + years with those in the 19–29 years and 30–49 years age groups constituting the majority (41% each). Those aged 70 + were only 4%. From the data, 73% of the respondents were found to be married while 25% were single and about 1% indicated that they were merely cohabiting with their partners. The majority (42.9%) of the respondents had a household size of 7–9 members, followed by 21.6% with household sizes of 4–6 members. A household size of 1–3 recorded the lowest percentage of 16.8%. In terms of educational levels of the respondents, only 10.6% were schooled up to the tertiary level, while 42.8% had no formal education and the rest had basic and secondary education. The data on respondents' occupation indicate that 39.2% of them were subsistence farmers and 10.8% were public/civil servants. A large proportion of the respondents were engaged in other economic activities, mainly artisanal employment (comprising masons, plumbers, electricians, hairdressers, etc.). The mean monthly incomes of respondents were between GHS480.00 and GHS3, 600.00.

### 4.2. Open Defecation Practices in the Wa Municipality

It was revealed that 49.8% of households had no form of toilet facility and were either using communal/public toilets or practicing open defecation. This result is in line with the 2010 Population and Housing Census [22] and Kosoe and Osumanu [7] which indicates that 47.8% and 52% of households, respectively, in the Wa Municipality had no toilet facilities in their homes, and therefore resort to free range in bushes, uncompleted structures, and open fields. Surprisingly, 84% of the respondents without home toilets had good understanding of the hygiene and health dangers associated with open defecation. The broader question seemed to be one of priorities: in the face of several unmet household needs and limited income, constructing a toilet facility does not seem to be a priority for many households. Financial constraints, which were mentioned by 94% of the respondents, translate into inability to procure construction materials and pay for labour. Respondents lamented over being already in debt over money borrowed for other things, such as food, weddings, or machinery for the farm and difficulties of generating money to pay such debts. This confirms the findings of Santah [23] when she concluded that people have expressed the pains of poverty which is displayed in the condition of some of their dwellings, dilapidated mud houses often with part of their roof falling off. A few (6%) of the respondents do not have toilet facilities in the house because they see no need for such (since there are places readily available for them to defecate in the open) and other reasons attributable to ancestral beliefs. A respondent said

*“I spend most of my time on my farm, so if I construct a toilet in my house it will be a waste. Because of this, I defecate in the bush before coming home. My late father used to practice the same.”*

The study revealed that 52% and 38% of female and male headed households, respectively, were without any form of toilet facility. The number of female headed households without toilet facilities is due, in part, to the fact that in traditional societies, like the Wa Municipality, women, no matter their status, do not see themselves as being responsible for the provision of household physical infrastructure. According to a female respondent,

*As a woman, my responsibility does not include building structures (including toilets) because I am a stranger and my late husband's family can send me away at any time. Maybe one of my sons will grow and construct a toilet for us.*

The results suggest that there is some relationship between educational background of respondents and ownership of toilet facilities as 65% of the households without formal education, and may be considered illiterates, did not own and use any household toilets. Also, 43% of the respondents who had attained primary education did not own and use home toilets. On the other hand, 88% of those who had tertiary education owned their own toilets. It is revealing that some uneducated persons perceive that it is only the educated who can own toilet facilities. A respondent expressed this view to a member of the research team:

*“My brother, looking at you, well-educated and dressed, I do not think you cannot construct a household latrine. But for me, my work will not fetch me enough money to construct a toilet facility.”*

The results again reveal that there is a relationship between occupation of respondents and ownership and usage of home toilet facilities. Out of 132 respondents who were farmers, only 6.3% owned a toilet facility. Moreover, only 5.2% artisans had household toilets. These respondents attributed their inability to own household toilet facility, to lack of finance, because their work does not generate enough income to enable them to construct such. According to a carpenter (household head),

*“I have 10 people in my household. I am taking care of them all. Imagine I hardly earn up to GHS150.00 a week. Where do you expect me to get money for toilet construction?”*

**Figure 3:**
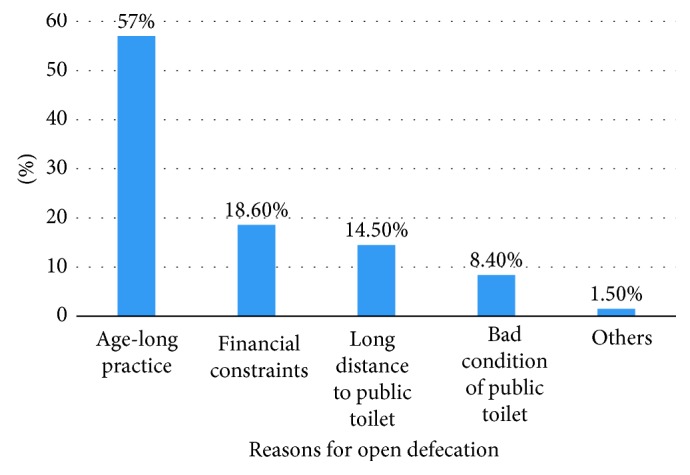
Causes of open defecation.

On the causes of open defecation in the Wa Municipality, 57% of the respondents indicated that the phenomenon is an age long practice handed down to them by their ancestors (Figure 3). In the words of a male respondent

*Our fore fathers were defecating openly without any toilet facility but lived over 100 years. How can you convince me that open defecation is not good?*

This finding collaborate Connell's [9] work in Peru, where respondents described open defecation as “*the most normal thing*.” However, the Municipal Environmental Health and Sanitation Officer attributed the incidence of open defecation to financial constraints. He told a research team member that

*“Last week, two gentlemen came to the Assembly to seek for financial assistance for the construction of a household latrine but the Assembly could not help them. We could only give them technical advice.”*

In the absence of home toilets, the use of public/communal facilities should be the practice. However, the inadequacy of such facilities within the Municipality leaves many people with no option than to defecate in the open. The Municipality currently has a total of 41 public toilets and most (58.5%) of them are found within the central business district (CBD), and its immediate surrounding communities with the rest dotted around other areas of the Municipality. Additionally, the bad state of public toilets was identified by the respondents as one of the reasons for people defecating in the open. The stench and heat emanating from public toilets often deter people from using the facilities. Nyonator [24] and Osumanu and Kosoe [5] implied this notion by opining that communal/public latrines already in existence needed continuous maintenance or users view them becoming hazardous facilities, thus encouraging the indiscriminate defecation by their intended users. Other causes of open defecation identified include nonenforcement of sanitation bye-laws, not being used to defecating in latrines or other toilet facilities, and requirements to pay for the use of public toilets. Reactions on payment to use public toilets were, however, mixed. Whiles some respondents complained of high fees charged, especially households with larger household sizes, others indicated that the amounts charged were adequate and affordable if only the facilities are kept clean always.

The Wa Municipal Assembly has enacted bye-laws to deal with sanitation related issues in the Municipality [25], including prosecution and fines. The results suggest that the bye-laws are not enforced since 86% of the respondents were not aware of the existence of bye-laws, especially in the rural areas. In spite of this finding, the Municipal Environmental Health and Sanitation Officer outlined the measures being enforced on open defecation as follows: fines, a six month ultimatum period for landlords to construct toilet facilities, formation of a sanitation task force to arrest people who defecate openly very early in the morning and late at night, and prosecution of offenders. He also mentioned the Criminal Code of 1960 (Act 29) and the Public Health Act (Act 851) as laws that are enforced alongside municipal bye-laws.

Respondents held diverse views about the role of culture in influencing open defecation. The results show that 68% of the respondents believed that cultural practices and beliefs influence where some people defecate. A public toilet attendant indicated that some people refuse to use the facility after 09 : 00 pm even though there are no charges after that hour. He explained that

*… it is believed that witches, wizards, and other bad spirits visit the toilet at night and as such woe unto anybody who is spotted by these spirits around those hours in the toilet.*

This agrees with studies by Belcher [13], Nawab et al. [26], Santah [23], and Osumanu and Kosoe [5] that cultural beliefs and practices prevent people from the usage of public/communal toilets. Contrary to this assertion, the religious and traditional leaders were of the view that cultural practices and beliefs cannot influence open defecation since it promotes the spread of diseases and pollutes the environment. A religious leader observed that religion has come to abolish all such traditional beliefs and practices since they are against defecating in the open.

From the responses of participants in this study, the following methods of open defecation were identified: the “*cat method*” (digging a hole in the ground and burying faeces after defecation); the “*flying method*” (defecating in polythene bags and throwing away in the open); and “*free range*” (defecating on refuse dumps, vacant plots, and in uncompleted buildings). Majority (48%) of the respondents in the rural parts of the Municipality indicated that in the absence of toilet facilities, the only option available is to dig a hole in the ground, defecate in it, and cover it thereafter. This is made possible by the availability of large tracks of unused land in rural communities. Also, this method of defecation is practised by farmers while on their farms. Covering the holes with faeces after defecation is an indication that those practising this method know the problems associated with inappropriate handling of human excreta. Whilst the ‘*cat method*' may be less dangerous in terms of its environmental and health effect, “*flying toilets*,” which is common in densely populated inner parts of the Municipality without toilet facilities, presents several challenges to residents [27]. Defecating on refuse dumps is a practice usually associated with children. Children are also allowed to defecate freely anywhere because their faeces are generally not regarded as “*harmful*.”

From this study, it is possible to identify the main sociocultural and economic factors that seem to determine open defecation in the Wa Municipality: age, education, sex, size of household, marital status, occupation, income, local taboos, traditional norms and beliefs, ownership of a toilet facility, and knowledge of the effects of open defecation. The mixed logit model [20] was used to establish the relationships between practising open defecation and this set of predictor variables (sociocultural and economic factors). The 11 predictor variables were selected to explain the dependent variable (open defecation).  Table 4 shows the logit model estimation of the sociocultural and economic determinants of open defecation in the Wa Municipality. Out of the total predictor variables of 11, six were significant at a 5% probability level. From the results, education, household size, occupation, income, traditional norms and beliefs, and ownership of a toilet facility significantly influence open defecation.

## 5. Discussion

Though this study shows that, in terms of sociocultural and economic factors, five of the selected determinants are not statistically significant in influencing open defecation; in general, the prevalence of the practice in the Wa Municipality makes residents vulnerable to its consequences. Through a lens of environmental health, dwellers of the Municipality are at high risk given that environmental sanitation is one of the major determinants of the incidence of infectious diseases. A large proportion (50%) of Ghana's health burden is caused by environmental-sensitive diseases such cholera, diarrhoea, and malaria [28], especially in northern Ghana where public provision for water and sanitation services are inadequate. Research on environmental health should be contextualised in terms of sociocultural and economic realities other than generalised indicators of disease burden. Using diverse sociocultural and economic indicators could help understand the important factors in designing any intervention. It also shows where interventions must first be targeted. For example, the results of this study show that sociocultural and economic vulnerabilities must first be addressed before other considerations.

Education is essential in human capital development and the appreciation of the need for environmental sanitation. The implementation of environmental health policies requires that both residents and environmental health officers have common grounds, and this requires some level of education [23]. The marginal effect of education in the logit model was observed to be negative and significant (*p*=0.073). The effect of education on open defecation is that households with educated heads are associated with a higher likelihood of not defecating in the open than those with noneducated heads (i.e., as the level of education increases, the likelihood of open defecation decreases). A household with an educated head has 18.5% higher chances of not defecation openly than their counterparts. This observation was expected since educated household heads can understand the effects of open defecation and the relevance of having a toilet at home. A higher level of education perhaps augments the income earning capacity of a household and its members, thereby expanding their capacity to construct a toilet facility and even adopt a better technology [29]. In this study, 65% of households with noneducated heads did not own home toilets and were likely to defecate openly.

The size of a household determines the practice of open defecation (*p* < 0.001, with a marginal effect of 0.40), meaning households with large sizes are 40% more likely to defecate in the open than those with smaller sizes. Responses from the household survey suggest that households with large sizes are more likely to defecate in the open. For instance, the survey discovered that 75% of households that have greater than nine members practiced open defecation, whilst 8% of those with 1–3 members did not. This suggests that heads of large households may be burdened with the need to cater for basic needs of all members, thereby reducing their capacity to construct toilet facilities at home. Household heads with many members claimed that the costs of building a toilet facility were high and hence not a necessary investment. Therefore, although they may be aware of the environmental and health benefits of owning toilets at home, their willingness to construct one will be negatively affected. Such households therefore choose to defecate in the open. This has serious implications for eradicating open defecation in the Municipality because 61.6% of the households covered in this study are composed of more than 6 members compared with the national average household size of 4.4 persons [15].

The results indicate a significant relationship between occupation and open defecation. Farmers constituted the single largest group (39.2%) of respondents, meaning that households whose heads are engaged in farming have a higher probability of defecating openly. The estimated marginal effect is 0.363, meaning that farmer-headed households have 36.3% more chance of open defecation than non-farmer-headed households. This is in line with the expectation of the research, since the occupation of an individual determines one's sources of income. Generally, farmers' incomes are low compared with those in other occupations. Particularly, in northern Ghana, rainfall conditions do not permit all-year farming and farmers are unemployed for a greater part of the year which affects their incomes. There is also the likelihood of farmers not having the urge to construct toilet facilities at home since they spend a greater part of their time on farms during the farming season.

As was expected, the income has a negative relationship with open defecation; the higher the income levels of a household head, the lesser the likelihood of its members practising open defecation. The coefficient of income in the logit model was negative and significant (*p*=0.001). Also, the marginal effect of income in the model was 0.343 meaning respondents with lower levels of income are 34.3% more likely to defecate in the open. This suggests that high income earners are more likely to own toilet facilities. This finding agrees with the *a priori* expectation that higher income will offer households the capacity to meet the cost of catering for basic needs including sanitation and agrees with the findings of other studies [5, 10].

The practice of open defecation is significantly influenced by belief systems (belief about sharing toilet facilities). In other words, the belief system of a particular household determines their use of shared toilet facilities (statistically significant at *p*=0.001). For instance, 57% of the respondents asserted that open defecation is an age long practice handed down to them by their ancestors. The findings revealed that households who believed (perceived) sharing toilet facilities with others, whether at home or communal places, may expose them to spiritual attacks prefer to defecate in the open even if they have access to toilet facilities. The results further illustrate that those households that belief in traditional norms are more likely not to build home toilets. Similar findings have been reported by Belcher [13], Cotton et al. [14], Action Aid [30], and Osumanu and Kosoe [5]. In this study, the marginal effect of traditional norms and beliefs was 0.333, indicating that households that respect traditional norms and beliefs are 33.3% more likely to practice open defecation. It is essential that these perceptions are noted in a study such as this that seeks to explore the determinants of defecation pathways of households. Eradicating open defecation entails that households should become aware that open defecation is not a healthy practice.

Although some members of households that own toilet facilities may practice open defecation, the results of this study indicate that ownership of a toilet facility is negatively related with the practice of open defecation (a coefficient of −0.635) and significant at 5% level (*p*=0.093). The estimated negative value and the significance of the variable (a marginal effect of 0.425) mean that households who do not own toilet facilities at home have a 42.5% greater chance of open defecation than those who own their own toilets. This is consistent with the research a *priori* expectation since having a toilet facility at home enables households to attend to nature's call conveniently at all times including rainy periods and odd hours. However, if home toilet facilities are not well maintained, some householders may opt to defecate in the open, especially where there are opportunities for them to do so.

## 6. Conclusion and Policy Implications

Open defecation is a serious sanitation issue most developing countries are battling with. Defecation is a natural urge and, subsequently, everyone will respond to it when the need arises. There are, however, clear differences of attitudes towards where people defecate. Even when poverty is being reduced and toilet facilities become available, cultural attitudes, social habits, and economic factors may impair people from the use or avoidance of infrastructure considered safe and hygienic by environmental and health standards. Understanding the sociocultural and economic factors underlying open defecation is therefore crucial for any policy aimed at eradicating the practice.

This study examined the sociocultural factors determining open defecation in the Wa Municipality in Ghana, using a mixed method approach. Unlike earlier studies conducted on open defecation in Wa and other parts of Ghana, this study has provided a comprehensive quantitative examination of the factors determining open defecation in the Municipality. Although the findings suggest that households have high knowledge of the environmental and health consequences of the practice of open defecation, several sociocultural and economic factors hinder them from using toilet facilities. These factors either make toilet facilities unavailable or inaccessible to households or they encourage people to defecate openly even when facilities for defecation are available and/or accessible. Using the logit estimation model [20], this study has identify six important factors—level of education, household size, occupation, income, traditional norms and belief, and ownership of a toilet facility—as being positively significant in determining open defecation. However, underlying many of the significant factors is how households can finance construction of home toilet facilities. Also, according to the findings, education is a great redeemer: it is one of the ways through which the final solution to open defecation practices can be found, especially when it comes to improving understanding and implementation of municipal environmental sanitation and health bye-laws as well as abolishing negative traditional attitudinal prejudices.

Given the enormity of open defecation in the Wa Municipality, new and innovative approaches of public education need to be considered. Such an approach should consider moving away from law enforcement and emphasise eradication of traditional practices which are inimical to using toilet facilities through the design of appropriate educational campaign messages. Religious and traditional bodies within the Municipality may be effective vehicles for channelling such messages. Also, the principle of credit financing may be considered in assisting households to construct home toilets. In this regard, there is a need to develop appropriate finance mechanisms, through partnerships with Municipal authorities and local financial institutions that ensure financial discipline and ability to recover the cost of investment. Finally, community-led initiatives that draw on the creativity and capacity of local people to take control of their change processes must be integrated into open defecation intervention programmes.

## Figures and Tables

**Table 1:** Study communities and sample sizes.

S. no.	Community	No. of households	Sample size	Sample selection interval
(1)	Dokpong	297	26	Every 11^th^ house
(2)	Sokpayiri	231	18	Every 13^th^ house
(3)	Kpongpaala	54	5	Every 11^th^ house
(4)	Konta	255	18	Every 14^th^ house
(5)	Zongo	78	7	Every 11^th^ house
(6)	Danko	224	18	Every 12^th^ house
(7)	Charia	450	37	Every 12^th^ house
(8)	Kpaguri	219	18	Every 12^th^ house
(9)	Chansa	144	11	Every 13^th^ house
(10)	Kumbiehe	96	7	Every 14^th^ house
(11)	Sombo	812	66	Every 12^th^ house
(12)	Boli	367	29	Every 13^th^ house
(13)	Jinkpang	97	7	Every 14^th^ house
(14)	Biihee	72	7	Every 10^th^ house
(15)	Dandafuro	462	37	Every 12^th^ house
(16)	Tampiani	38	5	Every 8^th^ house
(17)	Chegli	89	7	Every 13^th^ house
(18)	Piisi	171	15	Every 11^th^ house
(19)	Charingu	81	7	Every 12^th^ house
(20)	Jonga	167	15	Every 11^th^ house
(21)	Yibile	71	7	Every 10^th^ house
	Total	4,475	367	—

**Table 2:** Definitions of variables.

Variable	Definition/measurement	Expected outcome
Age	The total number of years from birth of a respondent	Positive
Education	The highest educational level attained by a respondent	Positive
Sex	This is a respondent being male or female	Positive
Household size	Total number of people in a household	Negative
Marital status	This is if a respondent is living with a spouse or not	Positive
Occupation	The type of economic activity engaged in by a respondent	Negative
Income	The level of a respondent's income	Positive
Local taboos	That is if a respondent indicates respect for taboos	Positive
Traditional norms and beliefs	That is if a respondent indicates respect for traditional norms and beliefs	Positive
Ownership of a toilet facility	If a respondent indicates the presence of a toilet facility at home	Positive
Knowledge of the effects of open defecation	That is if a respondent is aware the environmental and health consequences of open defecation	Positive

**Table 3:** Sociodemographic characteristics of respondents.

Characteristic	Description	Frequency	%		
Sex	Male	227	61.9		
	Female	140	38.1		

Age	19–29 years	153	41.7		
	30–49 years	153	41.7		
	50–69 years	46	12.5		
	70 + years	15	4.1		

Marital status	Married	269	73.3		
	Single	94	25.6		
	Others	4	1.1		

Occupation	Trading	69	19.7		
	Farming	144	39.2		
	Public/civil service	37	10.8		
	Others	117	33.3		

Household size	1–3	62	16.8		
	4–6	79	21.6		
	7–9	157	42.9		
	>9	69	18.7		

Formal education	None	157	42.8		
	Primary	52	14.2		
	JHS/middle	48	13.1		
	SHS/Tech/Voc.	67	18.3		
	Tertiary	39	10.6		
	Others	4	1.1		

*Monthly income (GHS)*	*N*	*Min*	*Max*	*Mean*	*Std. dev.*
	367	480.0	3,600.0	1,470.1	872.9

**Table 4:** Logit estimation of the determinants of open defecation.

Variable	Marginal effect	Std. error	*Z* value	*p* value	Coefficient
Age	−0.001	0.003	−0.41	0.684	−0.006
Sex	0.003	0.007	0.44	0.659	0.015
Education	0.185^*∗*^	0.103	1.79	0.073	−0.880
Household size	0.400^*∗*^	0.097	4.12	0.001	1.934
Household position	0.025	0.094	0.26	0.795	0.126
Marital status	0.002	0.005	0.46	0.643	0.011
Occupation	0.363^*∗*^	0.048	7.64	0.001	3.744
Income	0.343^*∗*^	0.090	3.79	0.001	−1.729
Traditional norms and beliefs	0.333^*∗*^	0.085	3.94	0.001	1.893
Local taboo	0.008	0.161	0.05	0.962	0.039
Ownership of a toilet facility	0.425^*∗*^	0.074	1.68	0.093	−0.635
Knowledge of the effects of open defecation	0.343	0.090	3.79	0.471	1.729

^*∗*^Significance at 5% level of confidence.

## Data Availability

Data were solely collected by authors, and they processed the data for analysis.
